# Non-uniqueness of factors constraint on the codon usage in *Bombyx mori*

**DOI:** 10.1186/s12864-015-1596-z

**Published:** 2015-05-06

**Authors:** Xian Jia, Shuyu Liu, Hao Zheng, Bo Li, Qi Qi, Lei Wei, Taiyi Zhao, Jian He, Jingchen Sun

**Affiliations:** Guangdong Provincial Key Laboratory of Agro-animal Genomics and Molecular Breeding, and Subtropical Sericulture and Mulberry Resources Protection and Safety Engineering Research Centre, College of Animal Science, South China Agricultural University, Guangzhou, 510642 People’s Republic of China; Guangzhou East Campus Lab Centre, Sun Yat-sen University, Guangzhou, 510006 People’s Republic of China; Innovative Drug Research Centre, Chongqing University, Chongqing, 401331 People’s Republic of China; Wageningen University, Wageningen, 6708 PG Netherlands

**Keywords:** *Bombyx mori*, Codon usage bias, Natural selection, Codon optimization

## Abstract

**Background:**

The analysis of codon usage is a good way to understand the genetic and evolutionary characteristics of an organism. However, there are only a few reports related with the codon usage of the domesticated silkworm, *Bombyx mori* (*B. mori*). Hence, the codon usage of *B. mori* was analyzed here to reveal the constraint factors and it could be helpful to improve the bioreactor based on *B. mori.*

**Results:**

A total of 1,097 annotated mRNA sequences from *B. mori* were analyzed, revealing there is only a weak codon bias. It also shows that the gene expression level is related to the GC content, and the amino acids with higher general average hydropathicity (GRAVY) and aromaticity (Aromo). And the genes on the primary axis are strongly positively correlated with the GC content, and GC3s. Meanwhile, the effective number of codons (ENc) is strongly correlated with codon adaptation index (CAI), gene length, and Aromo values. However, the ENc values are correlated with the second axis, which indicates that the codon usage in *B. mori* is affected by not only mutation pressure and natural selection, but also nucleotide composition and the gene expression level. It is also associated with Aromo values, and gene length. Additionally, *B. mori* has a greater relative discrepancy in codon preferences with *Drosophila melanogaster* (*D. melanogaster*) or *Saccharomyces cerevisiae* (*S. cerevisiae*) than with *Arabidopsis thaliana* (*A. thaliana*), *Escherichia coli* (*E. coli*), or *Caenorhabditis elegans* (*C. elegans*).

**Conclusions:**

The codon usage bias in *B. mori* is relatively weak, and many influence factors are found here, such as nucleotide composition, mutation pressure, natural selection, and expression level. Additionally, it is also associated with Aromo values, and gene length. Among them, natural selection might play a major role. Moreover, the “optimal codons” of *B. mori* are all encoded by G and C, which provides useful information for enhancing the gene expression in *B. mori* through codon optimization.

**Electronic supplementary material:**

The online version of this article (doi:10.1186/s12864-015-1596-z) contains supplementary material, which is available to authorized users.

## Background

Codon usage bias refers to differences of the occurrence frequency of synonymous codons in coding DNA. It is considered to be a product of mutation pressure and/or natural selection [1-4], and accounts for accurate and efficient translation, as well as mutation–selection–drift [5]. Codon bias analysis has been introduced into both prokaryotes and eukaryotes, such as *Escherichia coli* (*E. coli*), *Arabidopsis thaliana* (*A. thaliana*), and human beings [6-9], showing that codon bias has a high correlation to gene length, gene function, hydrophobicity of proteins, and the content of iso-acceptor tRNAs in genomes [9-12]. Hence, the analysis of codon usage can be used to study organism evolution and improve protein expression level [13-15].

The domesticated silkworm, *Bombyx mori* (*B. mori*), is a well-studied lepidopteran model system with rich genetic and molecular information of morphology, development, and behavior [13]. So far, the draft sequence for the genome of *B. mori* has been determined [16], and most studies of *B. mori* focus on the cloning, expression, and characterization of some genes or application as the bioreactor [17-19]. As we know, the analysis of codon usage is a good way to understand the genetic and evolutionary characteristics of *B. mori.* It can also help us to study the relationship between expression levels and codon usage bias since highly-expressed genes need abundant ribosomes and matching tRNAs for efficient translation. We have reported the codon usage bias of the mitochondrial genome in *B. mori* recently [20], however, the codon usage bias in the whole nuclear genome of *B. mori* is not well investigated in detail. Considering its great potential for expressing foreign proteins as a bioreactor, the codon usage bias of *B. mori* was examined here for codon optimization of genes.

## Results and discussion

### *B. mori* reveals a weak codon bias

**Figure 1:**
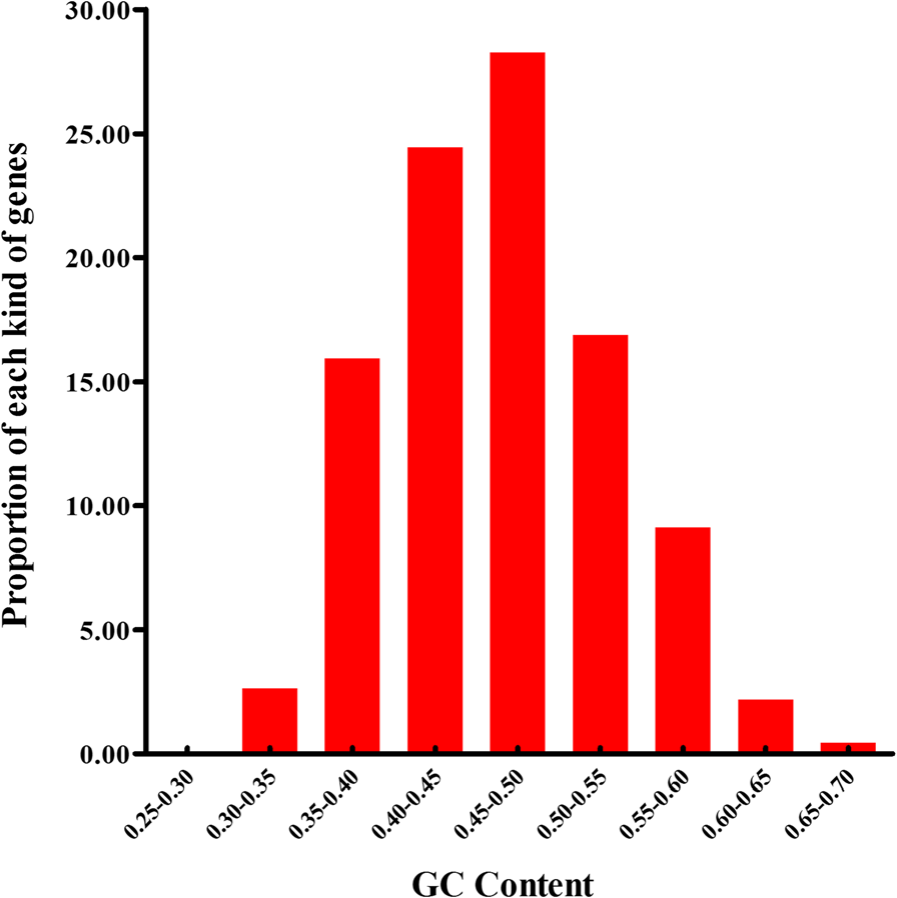
The distribution of GC contents in the CDS of *Bombyx mori.*

As shown in Additional file 1 and Table 1, the GC content for the total 1, 097 genes varies from 29.5% to 69.5%, with a mean value of 46.43%. The GC content of the total genes is distributed mainly between 40% and 50% (Figure 1). The greatest differences of GC content are found in the first and the third codon positions (51.92% and 48.40%, respectively), where most neutral mutations occur [21].

**Table 1:** **Means and standard deviations of GC, GC1, GC2, GC3, GC3s, ENc, CAI, A3s, T3s, C3s, G3s, Gravy, and Aromo of codons from** ***Bombyx mori***

**Class**	**Genes**	**Codons**	**GC (%)**	**GC1 (%)**	**GC2 (%)**	**GC3 (%)**	**GC3**_**S**_ **(%)**	**A3s (%)**	**T3s (%)**	**C3s (%)**	**G3s (%)**	**Gravy**	**Aromo**	**ENc**	**CAI**
Total	1097	363313	46.43 ± 6.72	51.92 ± 6.24	38.57 ± 6.02	48.40 ± 13.78	46.52 ± 14.37	34.28 ± 10.80	33.10 ± 9.43	32.37 ± 11.31	27.93 ± 8.86	−0.35 ± 0.36	0.09 ± 0.03	53.12 ± 5.47	0.76 ± 0.04
RP	67	11532	47.24 ± 4.60	52.14 ± 6.17	39.65 ± 5.47	49.21 ± 9.65	47.88 ± 10.10	30.78 ± 9.44	34.28 ± 6.17	33.08 ± 7.76	28.79 ± 7.35	−0.57 ± 0.28	0.06 ± 0.02	52.81 ± 5.15	0.80 ± 0.04

Note: RP indicates the ribosomal protein.

The effective number of codons (ENc) in *B. mori* ranges from 30.06 to 61.00, with an average of 53.12. As shown in Additional file 1, among the 1, 097 genes, only 5 genes reveal a high codon bias (ENc < 35). It indicates that *B. mori* exhibits a general random codon usage, without strong codon bias. Similarly, the relative synonymous codon usage (RSCU) values of 59 sense codons also support the conclusion that *B. mori* has a weak codon bias. As shown in Table 2, approximately half of the codons (28/59), denoted in bold lettering, are frequently used, such as GCU and AGA which encode Ala and Arg, respectively.

**Table 2:** **Codon usage of** ***Bombyx mori*** **genes (363,313 codons)**

**Amino acid**	**Codon**	**Total Count**	**RSCU**	**Amino acid**	**Codon**	**Total Count**	**RSCU**
**Ala**	**GCU**	8788	**1.37**	**Asn**	AAU	8005	0.95
	**GCC**	6997	**1.00**		**AAC**	8786	**1.05**
	GCA	5776	0.94	**Gln**	**CAA**	7174	**1.04**
	GCG	4791	0.68		CAG	6581	0.95
**Phe**	UUU	5896	0.84	**Ser**	AGU	3674	0.93
	**UUC**	8657	**1.15**		AGC	3882	0.94
**Gly**	**GGU**	6948	**1.22**		**UCU**	4628	**1.22**
	**GGC**	6590	**1.09**		UCC	3733	0.95
	**GGA**	7305	**1.25**		**UCA**	4413	**1.14**
	GGG	2670	0.45		UCG	3420	0.83
**Ile**	**AUU**	7139	**1.02**	**Thr**	**ACU**	5683	**1.16**
	**AUC**	7587	**1.13**		ACC	4634	0.90
	AUA	6226	0.85		**ACA**	5912	**1.23**
**Leu**	**UUA**	5362	**1.05**		ACG	3783	0.71
	**UUG**	6228	**1.17**	**Asp**	**GAU**	10241	**1.01**
	CUU	4403	0.88		GAC	10105	0.98
	CUC	5192	0.99	**Glu**	**GAA**	13868	**1.18**
	CUA	3554	0.68		GAG	9643	0.82
	**CUG**	6744	**1.22**	**His**	CAU	3805	0.91
**Pro**	**CCU**	5087	**1.19**		**CAC**	4585	**1.03**
	CCC	3689	0.83	**Lys**	**AAA**	13627	**1.09**
	**CCA**	4945	**1.12**		AAG	10889	0.91
	CCG	4154	0.84	**Arg**	**CGU**	3526	**1.12**
**Val**	**GUU**	6629	**1.11**		CGC	3318	0.95
	GUC	5830	0.93		CGA	2321	0.72
	GUA	5005	0.82		CGG	1700	0.47
	**GUG**	7235	**1.14**		**AGA**	5259	**1.74**
**Cys**	UGU	3008	0.89		AGG	3111	0.99
	UGC	3510	0.96				
**Tyr**	UAU	5246	0.85				
	**UAC**	7426	**1.14**				

Note: 1. Count indicates the number of codons.

2. The preferentially used codons are displayed in bold.

3. Hydrophobic and hydrophilic amino acids are listed on the left and right sides of the table, respectively.

In addition, most of preferentially used codons end with A/U (A/U-ended: G/C-ended=18:10). This phenomenon was also found in many other AT-rich species, such as *Pichia pastoris* (*P. pastoris*), *Saccharomyces cerevisiae* (*S. cerevisiae*), *Kluyveromyces lactis* (*K. lactis*), and *Plasmodium falciparum* (*P. falciparum*) [22,23].

### Effects of nucleotide composition in shaping codon bias

**Figure 2:**
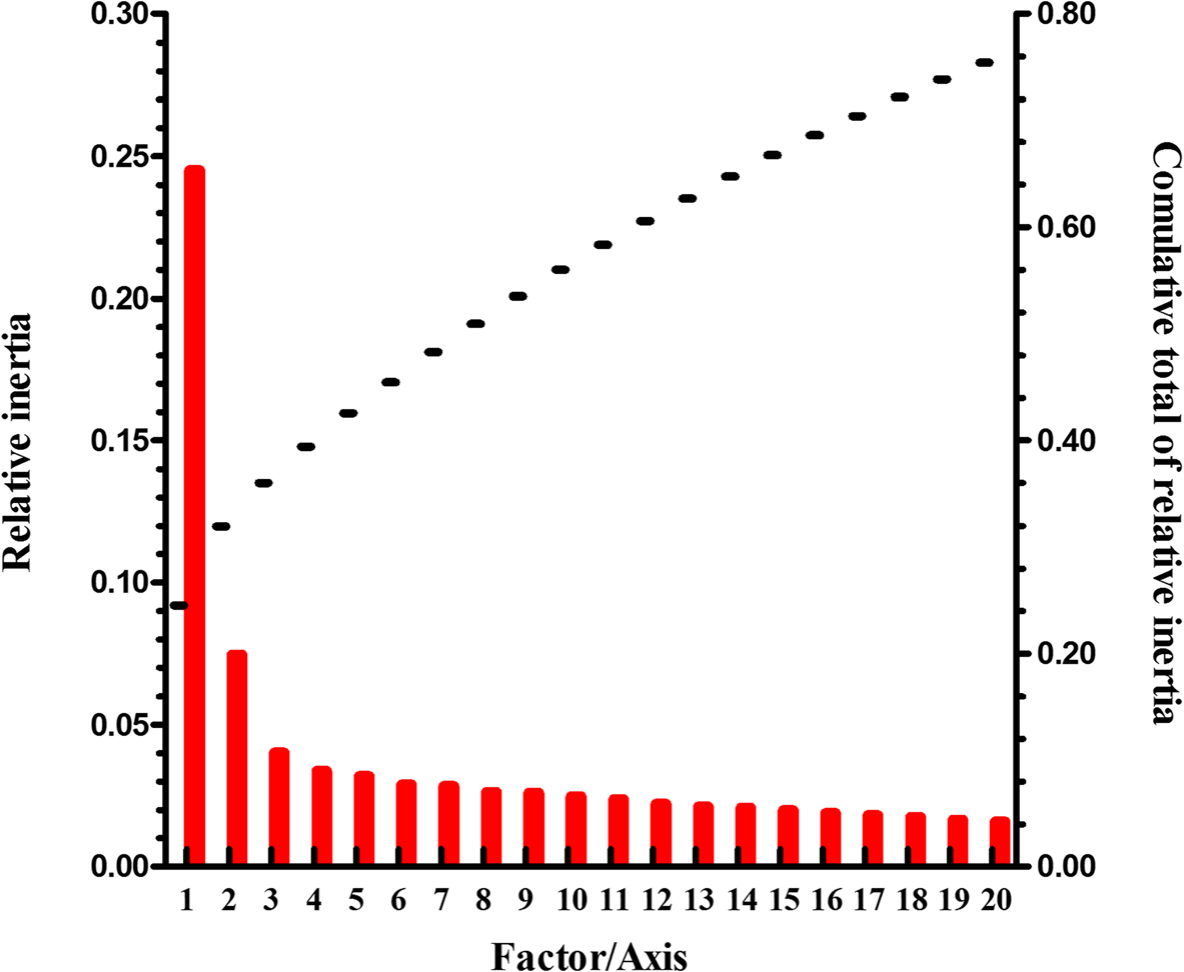
The relative and cumulative inertia of the first 20 factors from a correspondence analysis (COA) of the RSCU values.

Correspondence analysis of the RSCU values was used here, which removes the variation caused by the unequal usage of amino acids (although the degrees of freedom are reduced to 40 [24]), generating a first axis that explains 24.51% of the data inertia. The second axis explains 7.46%, while the next two axes respectively account for 4.02% and 3.39% of the data (Figure 2). Moreover, multivariable correlation analysis was introduced here to study the relationship between relative codon bias and nucleotide composition (Table 3).

**Table 3:** **Correlation coefficients between the positions of genes along the first two major axes with index of total genes’ codon usage and synonymous codon usage bias**

	**Gene length**	**GC**	**GC1**	**GC2**	**GC3**	**GC3s**	**A3s**	**T3s**	**C3s**	**G3s**	**Gravy**	**Aromo**	**ENc**	**CAI**	**Axis1**
**GC**	0.137**														
**GC1**	0.094**	0.699**													
**GC2**	0.132**	0.559**	0.392**												
**GC3**	0.128**	0.884**	0.418**	0.237**											
**GC3S**	0.125**	0.891**	0.434**	0.243**	0.998**										
**A3s**	−0.099**	−0.889**	−0.505**	−0.350**	−0.906**	−0.912**									
**T3s**	−0.154**	−0.787**	−0.334**	−0.230**	−0.902**	−0.900**	0.681**								
**C3s**	0.089**	0.834**	0.407**	0.201**	0.939**	0.943**	−0.861**	−0.834**							
**G3s**	0.135**	0.657**	0.272**	0.029	0.833**	0.828**	−0.707**	−0.763**	0.639**						
**Gravy**	0.166**	−0.021	−0.113**	0.020	0.041	0.034	−0.089**	−0.066*	0.003	−0.027					
**Aromo**	0.080**	−0.117**	−0.384**	−0.148**	0.075*	0.055	0.009	−0.060*	0.090**	0.031	0.235**				
**ENc**	0.079**	0.004	−0.046	0.018	0.043	0.037	0.057	−0.082**	−0.007	0.138**	0.045	0.100**			
**CAI**	−0.148**	0.282**	0.245**	−0.081**	0.300**	0.312**	−0.444**	−0.061*	0.434**	0.064*	−0.170**	−0.140**	−0.210**		
**Axis1**	0.030	0.420**	0.262**	0.122**	0.446**	0.446**	−0.444**	−0.367**	0.439**	0.343**	0.008	−0.020	0.055	0.229**	
**Axis2**	0.135**	−0.002	−0.165**	−0.033	0.108**	0.098**	0.148**	−0.336**	−0.028	0.320**	0.054	0.127**	0.184**	−0.636**	0.012

Note: ** p < 0.01. * p < 0.05.

**Figure 3:**
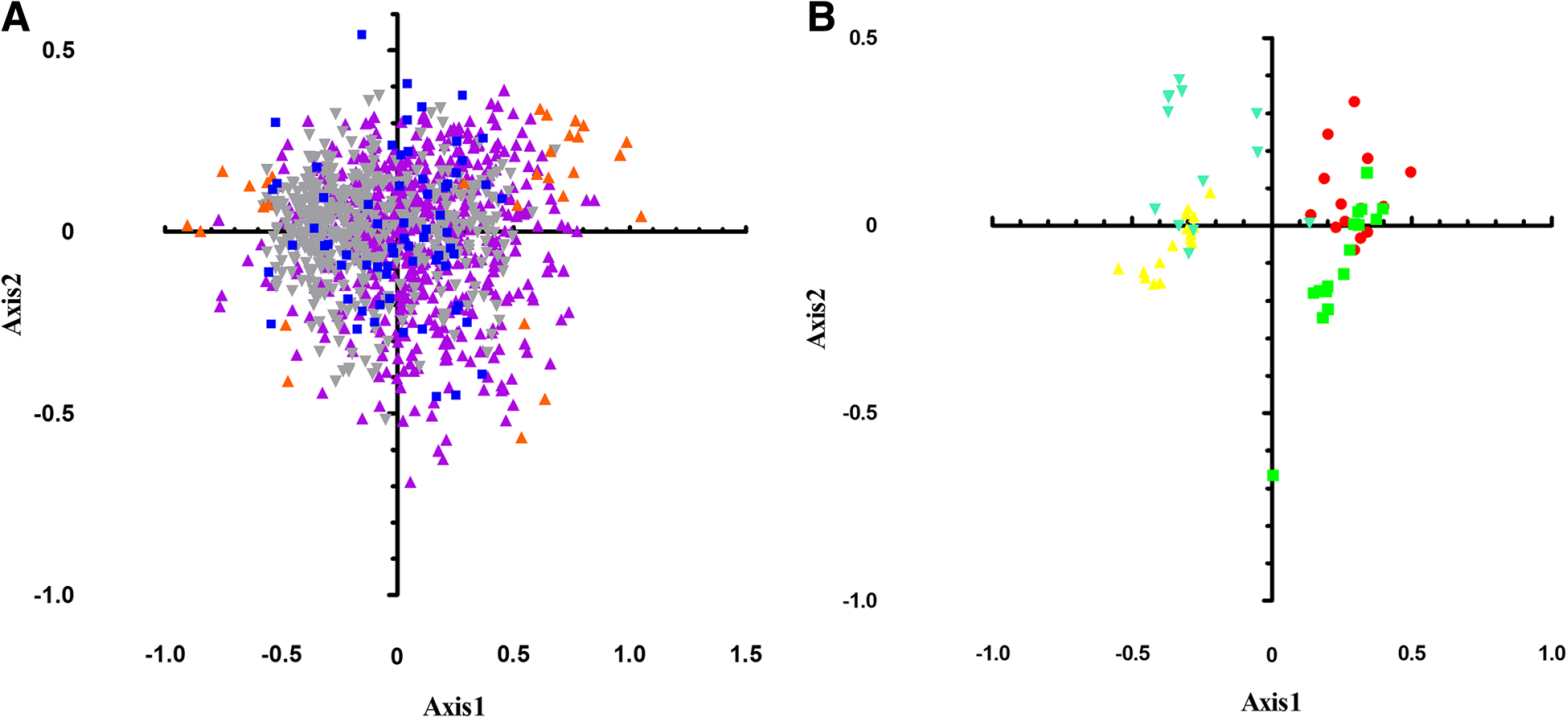
Correspondence analysis of RSCU for the total genes in *Bombyx mori.* **A)** Distribution of the total genes in *Bombyx mori* on the plane corresponding to the coordinates on the first and second principal axes was shown in Panel **A**. Orange triangles, purple triangles and gray triangles, indicate genes with a GC content higher than or equal to 60%, more than or equal to 45%, but less than 60% and less than 45%, respectively. Additionally, blue squares indicate the coordinates of ribosome on the first and second principal axes. **B)** Distribution of codons on the same two axes was shown in Panel **B**. Codons ending with A, U, C and G are shown in red, green, yellow, and blue, respectively.

Although the first axis can’t explain the whole variation, there is an obvious positive correlation between the first axis and G3s, C3s, and GC3s (r=0.343, 0.439, and 0.446, respectively, p < 0.01). However, the correlations between the first axis and A3s or T3s are negative (r=−0.444 and r=−0.367, respectively, p < 0.01). Then all the genes were classified into three categories by their GC content (GC < 45%, 45% ≤ GC < 60%, and GC ≥ 60%). As shown in Figure 3A, the position of each gene was marked along the first two major axes. Interestingly, the genes of GC < 45% are scattered at the left side of the first axis, while most of the genes with GC ≥ 60% are located at the right side of the first axis. The genes whose GC contents range from 45% to 60% are found in the middle of the plot. Additionally, almost all the ribosome genes are located in the range of GC ≥ 60%, implying that the expression level might be related with the GC content in *B. mori*.

On the overall consideration of Tables 1 and 3, it seems that the genes containing lower GC3s and GC content values tend to distribute at the left side of the first axis. Thus, we speculated that G/C-ending codons could be clustered at the positive side whereas A/U-ending codons gather at the negative side of first major axis. The corresponding distribution plot of synonymous codons ending with different bases along the two axes was implemented under the above mentioned assumption. The result indicates that the separation of codons on the first axis reflects the difference between the frequencies of A/U and C/G ending codons, while that on the second axis represents the frequency differences between A/G and U/C ending codons (Figure 3B), which is consistent with the above-mentioned hypothesis.

On the other hand, the ENc values show no significant correlation with the first axis (r=0.055, p > 0.05) or GC3s (r=0.037, p > 0.05) values, but a significant positive correlation with the second axis (r=0.184, p < 0.01) (Table 3).

The results above suggest that nucleotide composition has an effect on separating the genes along the first major axis, however, it might be not the main factor in shaping the codon bias.

### GC3s plays a minor role in shaping the codon bias of *B. mori*

**Figure 4:**
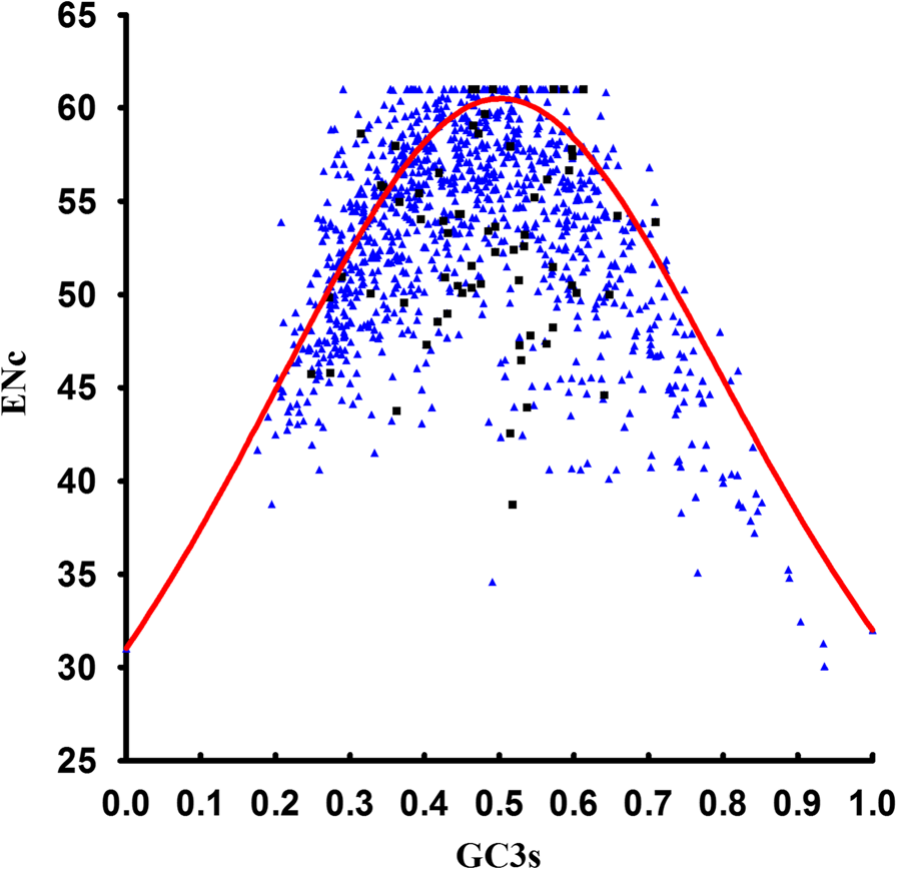
The ENc plotted against GC3s. ENc denotes the effective number of codons, and GC3s denotes the GC content on the third synonymous codon position. Black boxes, and blue triangles indicate ribosome genes and total genes, respectively. The red solid line represents the expected curve of positions of genes when the codon usage was only determined by the GC3s composition.

ENc-plot is an effective tool to study the codon usage patterns, and it was used here to explore the influence of GC3s on the codon bias of *B. mori*. As shown in Figure 4, most genes are located below the expected ENc-plot curve while only a small number of genes lay on or above the curve. It indicates that the conditional mutation might be a factor in shaping the codon bias but not the unique one.

**Figure 5:**
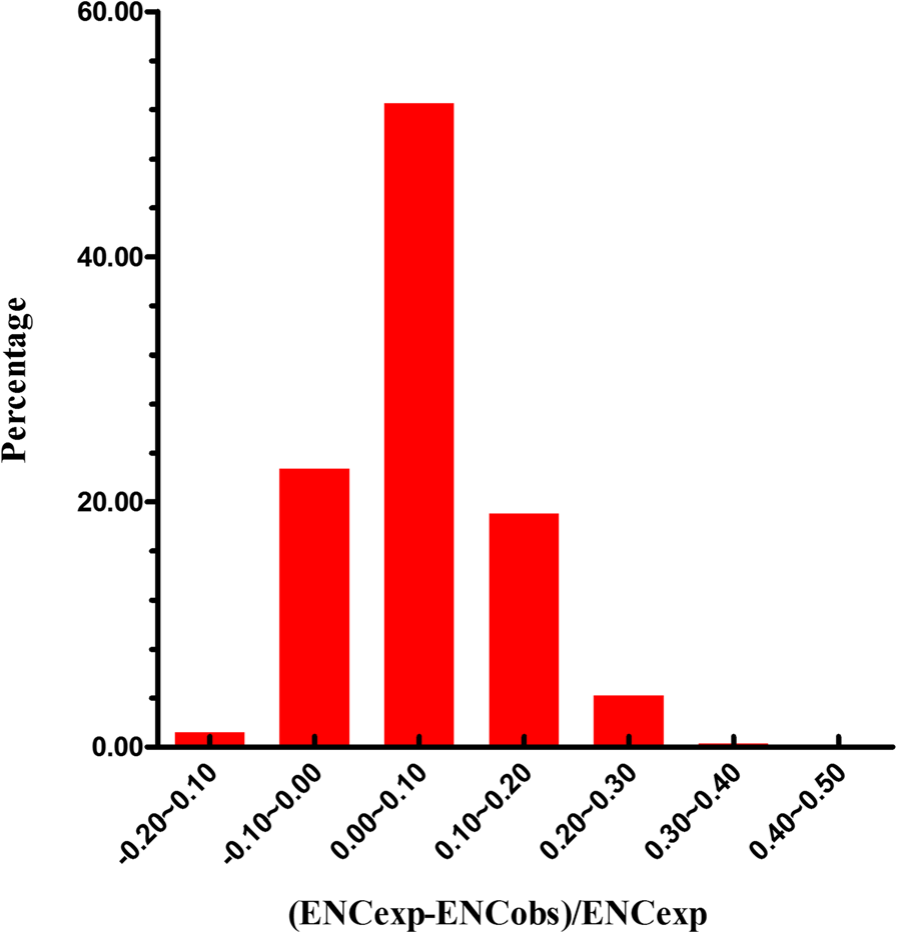
Frequency distribution of (ENCexp-ENCobs)/ENCexp.

We also estimated the difference between the observed and the expected ENc values using the plot of the frequency distribution of (ENCexp-ENCobs)/ENCexp in total genes (Figure 5). There was a similar single peak for each kind of genes. Peaks located within the 0 ~ 0.1 range of (ENCexp-ENCobs)/ENCexp values suggest that most actual ENc values are smaller than the ENc values from their GC3s. It is consistent with the results depicted in Figure 4, which shows that the difference in codon bias is dependent upon the differences in GC3s, thereby providing further evidence that GC3s works as a conditional mutational bias.

### Natural selection influences the codon bias as a major role

Although ENc plot can quantify the codon usage bias of synonymous codons, it is not sufficient to easily distinguish the main determinant factor between natural selection and mutational pressure within a species [25]. Therefore, a neutrality plot was implemented here.

**Figure 6:**
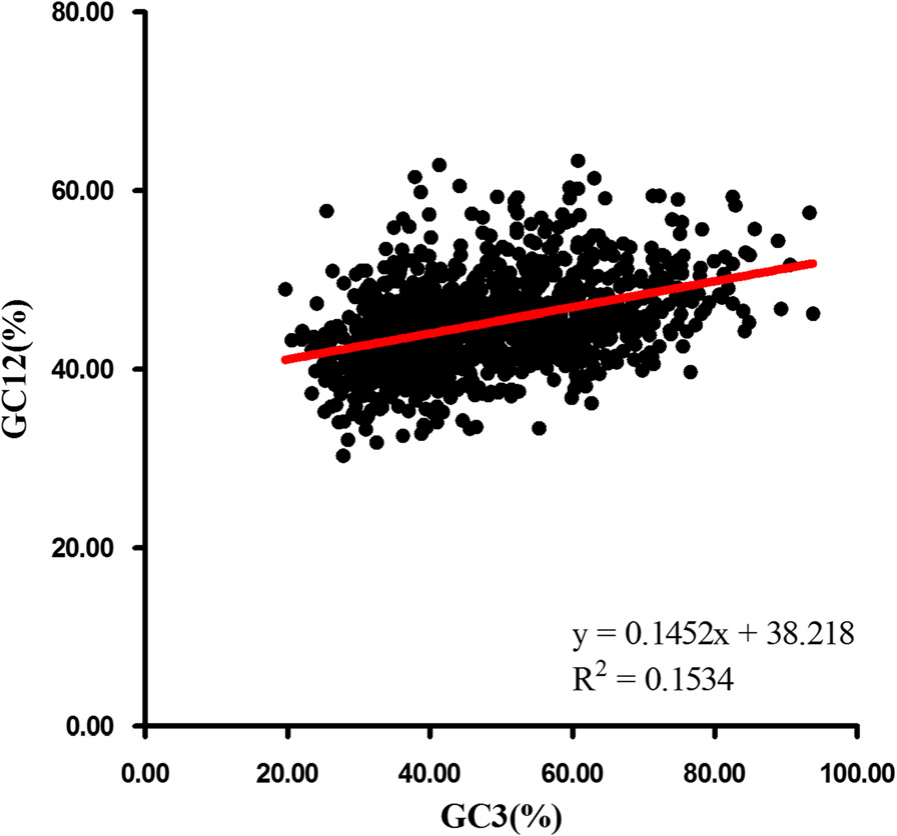
Neutrality plot analysis of the GC12 and that of the third codon position (GC3) for the entire coding DNA sequence of *Bombyx mori.* GC12 stands for the average value of GC content in the first and second position of the codons (GC1 and GC2). While GC3 refers to the GC content in the third position. The solid line is the linear regression of GC12 against GC3, R^2^=0.1534, P < 0.001.

The neutrality plot shows that the genes have a wide range of GC3 value distributions, ranging from 19.7% to 93.8% (Figure 6). There is a significant positive correlation between GC12 and GC3 (r=0.394, p < 0.01), suggesting that the effect of directional mutation pressure is present at all codon positions. Moreover, the slope of the regression line of the entire coding sequence is 0.1452. The results reveal that the effect of directional mutation pressure is only 14.52%, while the influence of other factors, for example natural selection, is 85.48% [26]. Accordingly, mutation bias only plays a minor role in shaping the codon bias, whereas natural selection probably dominates the codon bias.

### Codon usage bias in *B. mori* has a high correlation to aromaticity and gene length

In order to assess the relationship between the codon usage bias and hydrophobicity or aromaticity or gene length in *B. mori*, correlation analysis was performed. It could be observed from Table 3 that neither the Gravy values nor the Aromo values have significant correlation with GC3s. However, the Aromo values exhibit strongly positive correlation with the ENc values (r=0.100, p < 0.01), while the GRAVY values do not. The results indicate that the Aromo values are associated with the codon usage bias of *B. mori*.

The data in Table 3 also reveal that the gene length is positively correlated with the ENc values (r=0.079, p < 0.01), suggesting that gene length has a high correlation to the codon usage bias and might be also one of the factors contributing to the codon usage bias in genes.

### Effects of gene expression level

To explore the relationship between codon bias and gene expression level, correlation coefficients were calculated between the codon adaptation index (CAI) values and several other characteristics of the genes, including their position along the first major axis, the nucleotide composition, and the ENc values. Ribosome genes sequences were selected as the reference of highly expressed genes [15].

The results indicate that CAI, which represents gene expression level, shows significant negative correlation with the gene length (r=−0.148, p < 0.01), GC2 (r=−0.081, p < 0.01), A3s (r=−0.444, p < 0.01), T3s (r=−0.061, p < 0.05), Gravy (r=−0.170, p < 0.01), Aromo (r=−0.140, p < 0.01), and ENc (r=−0.210, p < 0.01). However, CAI shows obvious positive correlation with the first axis and the other nucleotide composition indices (i.e. GC, GC1, GC3, GC3s, C3s, and G3s, as shown in Table 3). The results above indicate that both nucleotide composition and gene expression levels are the major factors in shaping the codon usage bias of *B. mori*.

To statistically measure the relationship between the index of amino acid composition in *B. mori* and their codon bias, the correlation coefficients between the positions of the genes along the first four major axes with their indices of amino acid usage were analyzed using Spearman’s rank correlation analysis method and shown in Table 4.

**Table 4:** **Correlation coefficients between the positions of genes along the first four major axes with index of total genes’ amino acid usage**

	**Axis 1**	**Axis 2**	**Axis 3**	**Axis 4**	**CAI**	**Gravy**
**Axis 2**	0.017					
**Axis3**	0.022	−0.042				
**Axis 4**	0.024	0.049	−0.081**			
**CAI**	−0.216**	−0.208**	0.171**	−0.090**		
**Gravy**	0.327**	0.545**	0.479**	0.108**	−0.170**	
**Aromo**	0.208**	0.594**	−0.072*	0.245**	−0.140**	0.235**

Note: ** p < 0.01. * p < 0.05.

**Figure 7:**
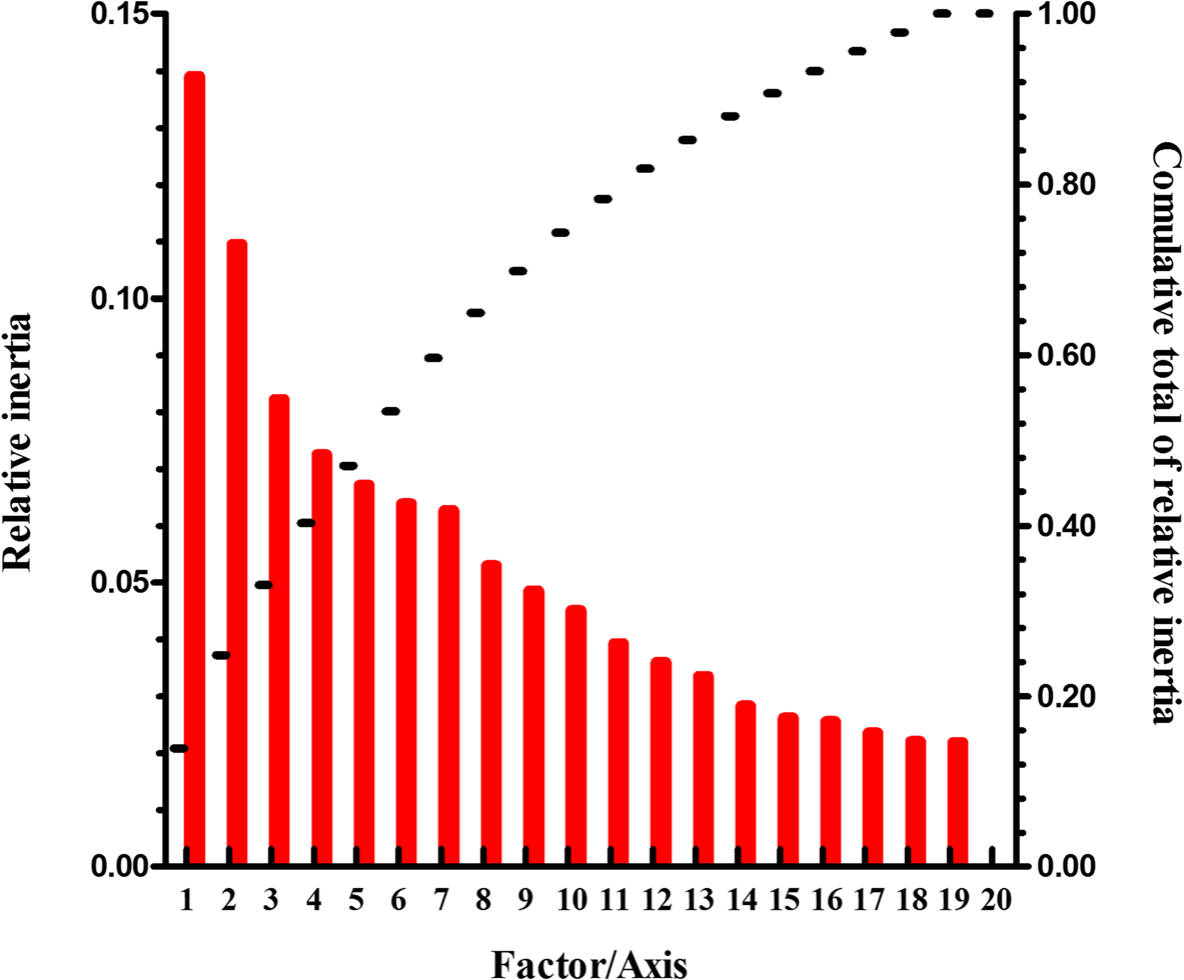
The relative and cumulative inertia of the first 20 factors from a correspondence analysis (COA) of the amino acid usage frequencies.

The first four axes generated by the correspondence analysis explain 40.31% of the amino-acid variation. And the first axis accounts for 13.90% of the variation in amino-acid usage (Figure **7**). The genes on these axes are all highly correlated with CAI, GRAVY score and Aromo value. The principle factor is negatively correlated with CAI (r=−0.216, p < 0.01), and is positively correlated with the GRAVY score and Aromo value (r=0.327, p < 0.01; r=0.208, p < 0.01, respectively). The second axis accounts for 10.95%, and is also correlated with the three indexes (r=−0.208, p < 0.01; r=0.545, p < 0.01; r=0.594, p < 0.01, respectively).

As in *E.coli* [27] where the most important trend in the amino-acid usage of *B. mori* is the usage of hydrophobicity, and the second important trend is the usage of CAI followed by the aromatic amino-acid. Taken all these together, it provides strong evidence for the inference that the effective selection of amino-acid for translational efficiency exists in *B. mori*.

In summary, the codon usage bias in *B. mori* is in some way or other, affected by nucleotide composition, mutation pressure, natural selection, and gene expression level. Additionally, it is also associated with Aromo values, and gene length. However, natural selection might play a major role in shaping codon usage variation, manifesting itself though weaker codon usage bias. The selection of amino-acid could also affect the translational efficiency in *B. mori*.

### Translational optimal codons of *B. mori*

In order to give a reference to enhance the expression level of important proteins with codon optimization, a two-way Chi-squared contingency test was used to compare the codon usage of different genes. Finally, the total putative optimal codons of *B. mori* are listed in Table 5. For the total genes group, the optimal codons all ended by G or C, and all amino acids—excluding Met and Trp—were identified by different numbers of codons. For example, three codons were identified for Ser, and two codons were identified for Ala, Gly, Leu, Pro, Val, Thr, and Arg. The remaining amino acids were identified by one codon.

**Table 5:** **Optimal codons of genes in** ***Bombyx mori***

**Amino acid**	**Codon**	**High**		**Low**		**Amino acid**	**Codon**	**High**		**Low**	
		**RSCU**	**N**	**RSCU**	**N**			**RSCU**	**N**	**RSCU**	**N**
**Ala**	GCU	0.64	288	1.66	315	**Asn**	AAU	0.34	130	1.39	549
	GCC *	1.80	818	0.46	88		AAC *	1.66	631	0.61	239
	GCA	0.29	133	1.65	313	**Gln**	CAA	0.44	174	1.43	370
	GCG *	1.27	575	0.23	43		CAG *	1.56	615	0.57	146
**Phe**	UUU	0.24	91	1.48	396	**Ser**	UCU	0.71	153	1.49	233
	UUC *	1.76	680	0.52	140		UCC *	1.71	366	0.44	69
**Gly**	GGU	0.65	240	1.49	291		UCA	0.42	90	1.77	276
	GGC *	1.91	703	0.58	113		UCG *	1.56	335	0.34	53
	GGA	0.75	277	1.55	303		AGU	0.34	72	1.40	219
	GGG *	0.69	254	0.39	76		AGC *	1.26	271	0.55	86
**Ile**	AUU	0.34	99	1.37	407	**Thr**	ACU	0.57	146	1.49	275
	AUC *	2.23	652	0.39	115		ACC *	1.68	433	0.43	79
	AUA	0.43	126	1.24	368		ACA	0.43	110	1.85	343
**Leu**	UUA	0.17	49	2.18	442		ACG *	1.32	339	0.23	43
	UUG	0.61	179	1.30	264	**Asp**	GAU	0.38	226	1.46	587
	CUU	0.30	88	0.89	181		GAC *	1.62	958	0.54	217
	CUC *	2.05	602	0.35	71	**Glu**	GAA	0.57	414	1.59	769
	CUA	0.27	78	0.75	152		GAG *	1.43	1036	0.41	197
	CUG *	2.60	764	0.53	108	**His**	CAU	0.38	97	1.44	196
**Pro**	CCU	0.48	122	1.46	204		CAC *	1.62	416	0.56	76
	CCC *	1.65	418	0.35	49	**Lys**	AAA	0.48	278	1.55	924
	CCA	0.36	91	1.87	261		AAG *	1.52	870	0.45	269
	CCG *	1.50	380	0.32	45	**Arg**	CGU	0.63	126	0.76	81
**Val**	GUU	0.41	144	1.59	363		CGC *	2.49	495	0.21	22
	GUC *	1.32	469	0.48	109		CGA	0.37	74	0.87	93
	GUA	0.36	126	1.26	289		CGG *	1.04	207	0.23	24
	GUG *	1.91	678	0.67	153		AGA	0.61	121	3.05	325
**Cys**	UGU	0.35	64	1.50	179		AGG	0.86	172	0.89	95
	UGC *	1.65	302	0.50	60	**Trp**	UGG	1.00	219	1.00	157
**Tyr**	UAU	0.29	100	1.40	322	**TER**	UGA	0.94	17	0.61	11
	UAC *	1.71	595	0.60	138		UAA	1.50	27	1.83	33
**Met**	AUG	1.00	507	1.00	359		UAG	0.56	10	0.56	10

Note: N is codon frequency, RSCU is relative synonymous codon usage. The codon usage of eleven genes (5% of the total number of genes) from the extremes of the principal were pooled. The codon usage of both pools was compared using a two-way Chi squared contingency test, to identify optimal codons. For the purposes of this test dataset with the lower ENc were putatively assigned as highly expressed. The codon usage and RSCU of both datasets is shown. Those codons that occur significantly more often (p < 0.01) in the highly biased dataset relative to the lower biased dataset are putatively considered optimal, and are indicated with a (*).

The optimization of codon usage allows improving the translational efficiency of foreign proteins by replacing the codons which are rarely found in the host organism [28], and it has been introduced into many heterologous systems [29-31]. As we found in this study, the optimal codons of *B. mori* are all ended by either G or C. This phenomenon is interesting and important to enhance the expression level of foreign proteins in *B. mori.*

### Comparison of codon preferences between *B. mori* and other model organisms

The ratio of codon frequency in *B. mori* was compared with five model organisms, including *A. thaliana*, *C. elegans, Drosophila melanogaster* (*D. melanogaster*), *S. cerevisiae*, and *E. coli*. The codon with a ratio of greater than 2, or less than 0.5, is defined as the indicative codon, of which usage frequency is markedly distinct from that of *B. mori.* As shown in Additional file 2, there are six and seven codons revealing distinct usage differences between *B. mori* and *D. melanogaster*, *S. cerevisiae,* respectively. However, there are only one, two or three codons with distinct usage between *B. mori* and *A. thaliana*, or *E. coli*, or *C. elegans*, respectively. It suggests that the discrepancy in codon preferences between *B. mori* and *D. melanogaster* or *S. cerevisiae* is relatively greater than that comparing with *A. thaliana*, or *E. coli*, or *C. elegans*. This finding implies that *B. mori* might have some advantages in expressing foreign proteins from certain organisms with fewer preferences in codon usage.

## Conclusions

After a series of analyses, the codon usage bias in *B. mori* is found to be weaker. And it is affected by nucleotide composition, mutation pressure, natural selection, and gene expression level. Additionally, it is also associated with Aromo values, and gene length. However, natural selection might play a major role in shaping the codon usage variation. In addition, it is also found that *B. mori* has a greater relative discrepancy in codon preferences in comparison with *D. melanogaster* or *S. cerevisiae* than with *A. thaliana*, *E. coli*, or *C. elegans*.

In summary, our analysis provides insights into the codon usage pattern in *B. mori* and is of the benefit to express foreign proteins in *B. mori* as a bioreactor.

## Methods

### Sequence collection

Accession numbers for a total of 1,213 reference sequences (RefSeq) of *B. mori* were obtained from Silkworm Genome Database (ftp://silkdb.org/pub/current/otherdata/Refseq/silkref.seq) (Downloaded on 1-Sep-2014). Coding DNA sequences (CDS) were downloaded from GenBank (http://www.ncbi.nlm.nih.gov). In these sequences, we only chose the CDSs without unidentified bases. To improve the quality of sequences and minimize sampling errors, genes without correct initiation and termination codons or with internal termination codons were ruled out. Additionally, only genes greater than 300 nucleotides in length were used for further analysis. As we only collected the CDSs from nuclear genome, 13 mitochondrial genes were excluded from the analysis. CDSs with gaps were also excluded. Finally, 1,097 CDSs were left for analysis, and each corresponds to a unique gene in *B. mori*.

### Indices of codon usage and synonymous codon usage bias

**GC3s** is a useful parameter for evaluating the degree of base composition bias, and represents the frequency of either a guanine or cytosine at the third codon position of synonymous codons, excluding Met, Trp, and stop codons.

**GRAVY** (General Average Hydropathicity) values are calculated as a sum of the hydropathy values of all the amino acids in the gene product divided by the number of residues in the sequence [32]. The more negative the GRAVY value, the more hydrophilic the protein, while the more positive the GRAVY value, the more hydrophobic the protein.

**Aromo values** denote the frequency of aromatic amino acids (Phe, Tyr, Trp) in the hypothetical translated gene product. The index and GRAVY value have been used to quantify the major COA trends in the amino acid composition of *E. coli* genes [27].

**RSCU** (relative synonymous codon usage) is the ratio of the observed frequency of codons relative to the expected frequency of the codon under a uniform synonymous codon usage. The RSCU value would be greater than 1.0 when the observed frequency is larger than the expected frequency [33].

**ENc** (Effective Number of Codons) values, varying from 20 to 61, are used to measure the magnitude of codon bias for individual genes, though it is worth noting that ENc values are affected by base composition [34]. A value of 20 indicates a gene with extreme bias using only one codon per amino acid, while a value of 61 indicates the absence of bias. In general, a gene is thought to possess strong codon bias if its ENc value is lower than 36 [35].

**CAI** (Codon Adaptation Index) values are often used to measure the extent of bias toward codons which are known to be preferred in highly expressed genes. With values ranging from 0 to 1.0, the higher the value, the stronger the codon usage bias and the higher the expression level. The set of sequences used to calculate CAI values in this study were the genes coding for ribosomal proteins in *B. mori* [35], so that it can provide an indication of gene expression level under the assumption that translational selection can optimize gene sequences according to their expression levels. These noted values and parameters were all utilized in this study.

All the indices of total genes and ribosomal protein genes are shown in Additional file 1, respectively.

### ENc-plot

The ENc-plot is a general strategy to investigate patterns of synonymous codon usage, where the expected ENc values are plotted against GC3s values. Expected ENc values were calculated according to Equation 1. In genes where codon choice is constrained only by a G + C mutation bias, predicted ENc values will lie on or around the GC3s curve, whereas if other factors such as selection effects are present, the values will deviate considerably below the expected GC3s curve [35].

1 $$ \mathrm{E}\mathrm{N}\mathrm{c}=2+\mathrm{S}+\left(29/\left({\mathrm{S}}^2+{\left(1\hbox{-} \mathrm{S}\right)}^2\right)\right) $$

S is the frequency of G + C (i.e. GC3s)

### Neutrality plot

A neutrality plot (GC12-GC3) [26] was used to estimate and characterize the relationships amongst the three positions in *B. mori* codons*.* A plot regression with a slope of 0 indicates no effects of directional mutation pressure (complete selective constraints), while a slope of 1 is indicative of complete neutrality.

### Determination of optimal codons

Based on axis 1 ordination, the top and bottom 5% of genes were regarded as the high and low datasets, respectively. Codon usage was compared using a two-way Chi-squared contingency test to identify optimal codons. The test dataset with the lower ENc values were putatively assigned as highly expressed, and those codons which occur significantly more often (p < 0.01) were defined as optimal codons [24].

### Correspondence analysis of RSCU

Correspondence analysis (COA) is a widely used method in the multivariate statistical analysis of codon usage patterns. Since there are a total of 59 synonymous codons (including 61 sense codons, minus the unique Met and Trp codons), the degrees of freedom was reduced to 40 in removing variations caused by the unequal usage of amino-acids while generating a correspondence analysis of RSCU [24].

### Software used

Mobyle server (http://mobyle.pasteur.fr), including CodonW (Ver.1.4.4) (http://mobyle.pasteur.fr/cgi-bin/portal.py#forms::CodonW), CHIPS (http://mobyle.pasteur.fr/cgi-bin/portal.py#forms::chips), and CUSP (http://mobyle.pasteur.fr/cgi-bin/portal.py#forms::cusp), were used to calculate useful indices of codon usage bias, such as GC, GC3s (G + C content at the third position of codons), silent base compositions (i.e. A3s, T3s, C3s, and G3s, which indicate the frequency that codons have an A, U, C, or G, respectively, at their synonymous third position), GRAVY values (general average hydropathicity), Armoro values (aromaticity), RSCU (relative synonymous codon usage), and ENc (effective number of codons). Similarly, a COA (correspondence analysis) was also performed.

CAI (codon adaptation index) and gene length were calculated using the CAI calculate server (http://genomes.urv.es/CAIcal). GC1, GC2, and GC3 values were also calculated to determine the GC content at the first, second, and third codon positions, respectively.

Together these indices allow for an assessment of the level to which selection has been effective in shaping codon usage [33]. Codon preferences of other organisms were downloaded from the Codon Usage Database (http://www.kazusa.or.jp/codon) for comparison.

### Statistical analysis

Correlations between codon usage variations amongst indices of codon usage were carried out using the multi-analysis software SPSS Version 22.0 (SPSS Inc. software, Chicago, Illinois, USA) and GraphPad Prism 5 (GraphPad Software, San Diego, California, USA).

## Additional files

Additional file 1:
**All the indices of total genes and ribosomal protein genes.**
Additional file 2:
**Comparison of codon preference between** ***B. mori*** **and other model organisms.** B/A, B/C, B/D, B/S, and B/E indicate the ratio between the frequency of codon usage in *B. mori* and *A. thaliana*, *C. elegans*, *D. melanogaster*, *S. cerevisiae*, as well as *E.coli*, respectively. Ratios larger than or equal to 2, or less than or equal to 0.5 are in bold.

## Abbreviations

CDS: Coding DNA sequences
GC12: Average value of GC content in the first and second position of the codons
GC3: GC content in the third position
GC3s: GC content on the third synonymous codon position
GRAVY: General average hydropathicity
Aromo: Aromaticity
RSCU: Relative synonymous codon usage
CAI: Codon adaptation index
ENc: Effective number of codons
COA: Correspondence analysis

## References

[1] Sharp PM, Li WH (1986). An evolutionary perspective on synonymous codon usage in unicellular organisms. J Mol Evol.

[2] Li WH (1987). Models of nearly neutral mutations with particular implications for nonrandom usage of synonymous codons. J Mol Evol.

[3] Sharp PM, Li WH (1987). The rate of synonymous substitution in enterobacterial genes is inversely related to codon usage bias. Mol Biol Evol.

[4] Morton BR (1998). Selection on the codon bias of chloroplast and cyanelle genes in different plant and algal lineages. J Mol Evol.

[5] Bulmer M (1991). The selection-mutation-drift theory of synonymous codon usage. Genetics.

[6] Lavner Y, Kotlar D (2005). Codon bias as a factor in regulating expression via translation rate in the human genome. Gene.

[7] Jia J, Xue Q (2009). Codon usage biases of transposable elements and host nuclear genes in *Arabidopsis thaliana* and *Oryza sativa*. Genomics Proteomics Bioinformatics.

[8] Sharp PM, Li WH (1986). Codon usage in regulatory genes in *Escherichia coli* does not reflect selection for ‘rare’ codons. Nucleic Acids Res.

[9] Duret L, Mouchiroud D (1999). Expression pattern, and surprisingly, gene length shape codon usage in Caenorhabditis, Drosophila, and Arabidopsis. Proc Natl Acad Sci U S A.

[10] Karlin S, Mrazek J (1996). What drives codon choices in human genes?. J Mol Biol.

[11] Zhou T, Gu W, Ma J, Sun X, Lu Z (2005). Analysis of synonymous codon usage in H5N1 virus and other influenza A viruses. Biosystems.

[12] Ikemura T (1985). Codon usage and tRNA content in unicellular and multicellular organisms. Mol Biol Evol.

[13] Arunkumar KP, Metta M, Nagaraju J (2006). Molecular phylogeny of silkmoths reveals the origin of domesticated silkmoth, *Bombyx mori* from Chinese *Bombyx mandarina* and paternal inheritance of *Antheraea proylei* mitochondrial DNA. Mol Phylogenet Evol.

[14] Henry I, Sharp PM (2007). Predicting gene expression level from codon usage bias. Mol Biol Evol.

[15] Banerjee R, Roy D (2009). Codon usage and gene expression pattern of *Stenotrophomonas maltophilia* R551-3 for pathogenic mode of living. Biochem Biophys Res Commun.

[16] Xia Q, Zhou Z, Lu C, Cheng D, Dai F, Li B (2004). A draft sequence for the genome of the domesticated silkworm (*Bombyx mori*). Science.

[17] Kobayashi N, Takahashi M, Kihara S, Niimi T, Yamashita O, Yaginuma T (2014). Cloning of cDNA encoding a *Bombyx mori* homolog of human oxidation resistance 1 (OXR1) protein from diapause eggs, and analyses of its expression and function. J Insect Physiol.

[18] Ihara H, Okada T, Ikeda Y (2014). Cloning, expression and characterization of *Bombyx mori* alpha 1,6-fucosyltransferase. Biochem Biophys Res Commun.

[19] Cai XY, Yu J, Yu HY, Liu YW, Fang Y, Ren ZX (2014). Core promoter regulates the expression of cathepsin B gene in the fat body of *Bombyx mori*. Gene.

[20] Wei L, He J, Jia X, Qi Q, Liang ZS, Zheng H (2014). Analysis of codon usage bias of mitochondrial genome in *Bombyx mori* and its relation to evolution. BMC Evol Biol.

[21] Zhang WJ, Zhou J, ZF L, Wang L, Gu X, Zhong Y (2007). Comparative analysis of codon usage patterns among mitochondrion, chloroplast and nuclear genes in *Triticum aestivum* L. J Integr Plant Biol.

[22] Peixoto L, Fernandez V, Musto H (2004). The effect of expression levels on codon usage in *Plasmodium falciparum*. Parasitology.

[23] Zhao X, Huo KK, Li YY (2000). Synonymous codon usage in *Pichia pastoris*. Chin J Biotechnol.

[24] Peden JF. Analysis of codon usage. PhD thesis. Nottingham University, Department of Genetics; 1999

[25] Kawabe A, Miyashita NT (2003). Patterns of codon usage bias in three dicot and four monocot plant species. Genes Genet Syst.

[26] Sueoka N (1988). Directional mutation pressure and neutral molecular evolution. Proc Natl Acad Sci U S A.

[27] Lobry JR, Gautier C (1994). Hydrophobicity, expressivity and aromaticity are the major trends of amino-acid usage in 999 *Escherichia coli* chromosome-encoded genes. Nucleic Acids Res.

[28] Condon A, Thachuk C, Iliopoulos C, Smyth W (2011). Efficient codon optimization with motif engineering. Combinatorial algorithms.

[29] Peng RH, Yao QH, Xiong AS, Cheng ZM, Li Y (2006). Codon-modifications and an endoplasmic reticulum-targeting sequence additively enhance expression of an *Aspergillus* phytase gene in transgenic canola. Plant Cell Rep.

[30] Ko HJ, Ko SY, Kim YJ, Lee EG, Cho SN, Kang CY (2005). Optimization of codon usage enhances the immunogenicity of a DNA vaccine encoding mycobacterial antigen Ag85B. Infect Immun.

[31] Song HF, Li GH, Mai WJ, Huang GP, Chen KP, Zhou YJ (2014). Codon optimization enhances protein expression of *Bombyx mori* Nucleopolyhedrovirus DNA Polymerase in *E. coli*. Curr Microbiol.

[32] Kyte J, Doolittle RF (1982). A simple method for displaying the hydropathic character of a protein. J Mol Biol.

[33] Sharp PM, Li WH (1987). The codon adaptation index–a measure of directional synonymous codon usage bias, and its potential applications. Nucleic Acids Res.

[34] Novembre JA (2002). Accounting for background nucleotide composition when measuring codon usage bias. Mol Biol Evol.

[35] Wright F (1990). The ‘effective number of codons’ used in a gene. Gene.

